# Chronic Comorbidities and Failure of Non-Operative Management in Adhesive Small Bowel Obstruction: Results of Analysis of National Inpatient Data from the United States

**DOI:** 10.3390/jcm14175989

**Published:** 2025-08-25

**Authors:** Gal Malkiely, Maya Paran, Miri Elgabsi, Boris Kessel

**Affiliations:** 1 Division of Surgery, Hillel Yaffe Medical Center, Hadera 38100, Israel; miriv42@gmail.com; 2 Department of Pediatric Surgery, Schneider Children’s Medical Center, Petah Tikva 4920235, Israel; paran.maya@gmail.com; 3 Rappaport Faculty of Medicine, Technion-Israel Institute of Technology, Haifa 31096, Israel

**Keywords:** adhesive small bowel obstruction, non-operative management, age, comorbidities, failure, predictive factor

## Abstract

Background: Adhesive small bowel obstruction (ASBO) is a common and challenging surgical condition. In the absence of peritonitis, bowel ischemia, or clear surgical indicators on CT imaging, the initial management is typically non-operative. While clinical and radiological factors influencing non-operative management (NOM) are well described, the role of age and chronic health conditions remains less well defined. The primary aim of this study was to evaluate the incidence of NOM failure in patients with various comorbidities. Methods: This study utilized data from the National Inpatient Sample to analyze cases of ASBO between 2016 and 2019. Collected data included demographics, diagnosis, presence of chronic health conditions (diabetes mellitus, congestive heart failure, chronic kidney disease, chronic pulmonary diseases, peripheral vascular disease), length of hospital stay, and mortality. Patients were divided into two groups: Group A (18–65 years) and Group B (>65 years). We compared demographics comorbidities, NOM failure rates, and mortality between the groups. Univariate analysis was performed to assess age and comorbidities and risk factors for NOM failure in each group, followed by multivariable analysis within each group. Results: A total of 1,611,099 admissions with ASBO were identified in the NIS database; 63.03% were females. The failure rate of NOM in patients without comorbidities was 21%, compared to 26.5% in patients with one or more comorbidities. In Group A, 20% of patients required surgery, compared to 26.2% of patients in Group B (*p* = 0.001). Conclusions: Being aged over 65 and the presence of chronic health disease, excluding diabetes mellitus, are independent predictors of NOM failure in patients with ASBO. The presence of multiple comorbidities further increases the risk of NOM failure.

## 1. Introduction

Adhesive small bowel obstruction (ASBO) is a common and clinically significant condition in general surgical practice, representing approximately 20% of all surgical emergencies [1]. The reported incidence following abdominal surgery varies between 2% and 13%, depending on the type of surgery, patient population, and length of follow-up [2,3]. The highest incidence is reported after pelvic procedures, particularly those involving colorectal and gynecological surgery [4,5]. In the absence of indications for immediate surgery, most patients are initially treated with non-operative management (NOM), which typically includes bowel rest, nasogastric decompression, fluid resuscitation, and, in select institutions, water-soluble contrast administration. However, the true rate of NOM failure rates remains unclear, with recent series reporting rates ranging from 20 to 45% [6,7,8]. The high burden of ASBO is reflected not only in its frequency but also in its considerable impact on patient morbidity, healthcare utilization, and resource utilization. Despite decades of accumulated clinical experience, the management of ASBO remains both challenging and controversial, despite there being multiple published guidelines, consensus statements, and institutional protocols [9,10].

A central point of ongoing debate concerns the timing and selection of operative versus non-operative management (NOM). Surgeons who support early surgical intervention argue that prompt operation may mitigate the risk of intestinal ischemia and the subsequent need for bowel resection—events that are associated with significant morbidity, prolonged recovery, and increased mortality. Early surgery may also reduce the systemic inflammatory response and the likelihood of serious complications. Conversely, NOM has been shown to result in a shorter hospital stay in patients who respond favorably, thereby reducing immediate healthcare costs and avoiding operative risks. However, conservative treatment carries its own drawbacks, including a higher recurrence rate of ASBO and a shorter interval to re-admission, although the overall risk of requiring surgery in future episodes appears unchanged.

These contrasting management outcomes underscore the necessity for individualized decision-making in which patient-specific clinical factors, comorbidities, and physiological reserve are carefully weighed against the potential risks and benefits of each approach. As such, optimal management of ASBO requires a nuanced strategy that balances timely intervention against the benefits of avoiding unnecessary surgery, while ensuring that deterioration is recognized and acted upon without delay.

Several epidemiological, clinical, and radiological factors have been identified as predictors of NOM failure. With no doubt, the most important element is the overall clinical profile of the patient. The presence of increased amounts of intestinal contents drained via nasogastric tube, the new appearance of abdominal pain, or its persistence associated with a bloated abdomen will most likely increase the likelihood of requiring operative intervention [7]. In addition, the history of multiple previous laparotomies is considered a well-described factor of NOM failure in these patients [11,12]. Radiological factors also play a critical role in decision-making process. Poor bowel wall enhancement and a high level of obstruction on CT imaging are independently associated with failure of non-operative management, as these findings may indicate ischemia or complete bowel obstruction [13]. For example, some studies have shown that advanced age, particularly patients over 65 years, is associated with a significantly higher risk of NOM failure and an increased likelihood of requiring surgical intervention [14,15]. In contrast, the impact of chronic health conditions on the success of NOM in ASBO is less well defined. While various studies suggest that chronic comorbidities are associated with a poorer response to conservative management [16,17], these studies are often limited by small sample sizes and heterogeneous patient populations. Theses gaps in the literature prompted us to conduct a study using a large U.S. national database. The primary aim of the study was to evaluate the incidence of NOM failure rates in patients with ASBO and to identify potential predictive factors, with a focus on age and the presence of chronic health conditions in patients.

## 2. Patients and Methods

### 2.1. Study Design

This retrospective study utilized data from the National Inpatient Sample (NIS) to investigate ASBO in adults aged 18 years and older in the United States. The NIS is the largest publicly accessible all-payer inpatient care database, encompassing data from over seven million hospital stays. Data from 1 January 2016 and 31 December 2019 were included in the analysis. The NIS database is publicly accessible at https://hcup-us.ahrq.gov/db/nation/nis/nisdbdocumentation.jsp (accessed on 17 August 2025).

### 2.2. Patient Selection

Patients with a diagnosis of ASBO were identified using the International Classification of Diseases, Tenth Revision (ICD-10) codes. Inclusion criteria included all patients aged 18 years and older with a diagnosis of AI, according to the ICD-10 code of K56.5. Patients were stratified into two groups: Group A (aged 18–65 years) and Group B (≥65 years).

### 2.3. Data Collection and Analysis

Collected variables included patient demographics (age, gender), diagnosis, presence of chronic health conditions, length of hospital stay, and mortality. Chronic health conditions were defined according to the established literature and included diabetes mellitus (DM), congestive heart failure (CHF), chronic kidney disease (CKD), chronic pulmonary diseases (CPDs), and peripheral vascular disease (PVD). Comparisons between Groups A and B were conducted for demographic characteristics, existing comorbidities, rates of NOM failure, and mortality. Univariate analyses of age and chronic health conditions as potential risk factors for NOM failure were performed separately for each group. Variables found to be significant in univariate analysis were subsequently included in a multivariable logistic regression model to identify independent predictors of NOM failure in each age group. Failure of non-operative management (NOM) was defined as the need for surgical intervention during the same hospital admission. Surgical procedures were identified using ICD-10-PCS codes associated with operative treatment for small bowel obstruction. Time to surgery and specific clinical deterioration parameters could not be assessed due to the structure of the NIS data set.

### 2.4. Statistical Analysis

A comparison between Groups A and B was performed for baseline and studied variables. A Shapiro–Wilk test was performed for each continuous variable to determine whether it was normally distributed. Accordingly, continuous variables were compared using the independent sample *t*-test or the Mann–Whitney test, as required. The Pearson X2 test or Fisher’s exact test was performed for categorical variables, as needed. Following the univariate analysis, a multivariable logistic regression model for NOM failure rates was constructed to determine risk factors in each study group separately. Statistical significance was considered as a two-tailed *p*-value of 0.05 or less. All analyses were performed using SPSS software version 29. 

Due to the limitations of the NIS database, variables such as symptom duration, clinical presentation, or surgical indication were unavailable. Missing data were minimal for included variables, and complete case analysis was used. Potential unmeasured confounders could not be adjusted for.

## 3. Results

During the study period, 1,611,099 admissions were identified in the NIS database with a diagnosis of ASBO. Of these, 595,504 (36.97%) were male and 1,015,595 (63.03%) were female.

Group A (ages 18–65 years) included 956,865 (59.4%) admissions with a mean age of 48.48 years (St. deviation 12.084) and a mean length of hospital stay of 6.16 (St. deviation 8.452) days.

Group B, (age > 65 years) included 654,234 (40.6%) admissions with a mean age of 75.72 (St. deviation 6.999) years, and the mean length of hospital stay was 7.88 days (St. deviation 8.093).

Observed mortality was statistically significant lower in Group A compared to Group B (0.75% vs. 3.13%, *p* < 0.01).

Regarding operative interventions, 20.0% of admissions in Group A (191,090 cases) required surgery, compared to 26.2% in Group B (171,390 cases). This difference highlights the increased likelihood of surgical treatment among elderly patients.

It should be noted that NOM failure rates (as stratified by comorbidity status) and operative intervention rates (as stratified by age group) are based on overlapping but non-identical patient subgroups, which explains the slight variation between the reported percentages.

We evaluated the presence and distribution of chronic health conditions across both groups (Table 1).

As shown in Table 1, the prevalence of chronic comorbidities differs significantly between age groups.

To better understand how these conditions influence the success or failure of non-operative management (NOM), Table 2 presents NOM failure rates stratified by comorbidity and age group.

To further evaluate the contribution of each comorbidity to the likelihood of operative intervention, we performed a multivariable logistic regression analysis to identify independent risk factors for surgical interventions (Table 3).

Notably, diabetes mellitus was not found to be an independent predictor of NOM failure in the multivariable model. While included in the analysis, it did not reach statistical significance and was therefore not emphasized in the conclusions.

To assess the additive effect of multiple comorbidities on NOM failure, we analyzed the data by the total number of chronic conditions per patient. Table 4 presents the risk of NOM failure stratified by cumulative comorbidity burden.

In both groups, each additional comorbidity significantly increases the risk of requiring surgery. In Group A (younger patients), one comorbidity increased the odds by 38.2%, two by 33.7%, three by 46.4% and four and more comorbidities increase the risk for surgery in 56.2%

In Group B (older patients), one comorbidity increased the odds by 14.2%, two by 13.6%, and three by 21.3%. Four or more comorbidities increased the risk for surgery by 14.4%.

## 4. Discussion

Multiple studies and guidelines have been published in an effort to optimize the management of patients with ASBO [18,19]. Current evidence suggests that, in the absence of signs of peritonitis or bowel ischemia, approximately 80% of patients may be safely managed non-operatively [20]. An awareness of factors associated with NOM failure is critical for clinical decision-making and patient selection.

The primary predictors of NOM failure are typically clinical, including persistent abdominal pain, fever, abdominal distension, or worsening physical examination findings [18]. Radiological factors also play a key role and may include intestinal wall thickening, reduced bowel wall enhancement, high-grade obstruction, or the presence of peritoneal free fluid [21,22]. Despite this, determining the need for surgical intervention remains challenging, even for experienced acute care surgeons. Several scoring systems have been proposed to aid decision-making, which emphasize the importance of integrating clinical and radiological findings [23,24]. In contrast, the influence of age and chronic health conditions on NOM outcomes has been less thoroughly evaluated, and the existing literature on this subject is inconsistent. We believe that understanding the role of these factors is important, as it may improve patients’ stratification and help guide the timing and duration of conservative treatment. Our findings align with previous studies that identified increased age as a predictor of NOM failure in adhesive small bowel obstruction [14,16]. Notably, in our study, patients over the age of 65 years had a significantly higher risk of requiring surgery (38.7%), exceeding some previously reported rates. Based on this large data set, we suggest that while NOM remains appropriate in the absence of peritonitis or strangulation, the threshold for surgical intervention should be significantly lower in older adults.

The impact of chronic health conditions on the outcome of conservative treatment has also been underexplored. Some studies have suggested an association between comorbidities and increased risk of NOM failure, though statistical significance was not always established. For example, Al-Wageeh et al. reported that patients with diabetes mellitus had a fourfold higher failure rate of NOM, although this association was not statistically significant in multivariable analysis [25]. Similarly, Cho et al. observed comparable trends in patients with diabetes mellitus and hypertension [26]. In contrast, Maraux reported that a Charlson comorbidity index score ≥ 4 was associated with increased risk of NOM failure [24], whereas another study involving 777 patients found that a high Charlson comorbidity index predicted major complications but not NOM failure specifically [27]. Our study adds important insights to this debate. In our large national cohort, each of the examined chronic health conditions, except for diabetes mellitus, was independently associated with a higher likelihood of NOM failure.

Chronic health disease can significantly complicate the conservative management of adhesive ileus by increasing the risk of adverse outcomes, prolonging recovery, and reducing the likelihood of successful non-operative resolution. Patients with chronic comorbidities—such as cardiovascular disease, chronic kidney disease, diabetes, or chronic respiratory insufficiency—are more susceptible to fluid and electrolyte imbalances, hypovolemia, and systemic complications during ileus due to impaired physiological reserve and altered homeostatic mechanisms. These patients are also at higher risk for developing complications such as sepsis, multi-organ dysfunction, and poor wound healing if surgical intervention becomes necessary

However, chronic disease may limit the tolerance for prolonged bowel rest and fasting, increase the risk of aspiration, and complicate fluid management due to underlying cardiac or renal dysfunction. Additionally, the presence of chronic illness may mask or mimic symptoms of clinical deterioration, making timely recognition of failed conservative management more challenging [28]. Furthermore, we demonstrated a clear cumulative effect: our results demonstrate that the risk of failure of NOM increased with the number of comorbidities. This finding highlights the importance of considering comorbidity burden when evaluating patients for conservative versus surgical management.

## 5. Limitations

This study has several limitations that should be underlined. First, it is a retrospective analysis based on registry data, which lacks detailed clinical and radiological information, such as the time from symptom onset to hospital presentation, the timing and type of surgery, and other key decision-making parameters. Additionally, the NIS database does not include granular clinical assessments, which limits the ability to incorporate established predictive scoring systems. Given the multi-center nature of the registry, variability in institutional protocols and surgeon decision-making may introduce heterogeneity and bias. Finally, every patient may have more than one admission, but the database structure is limited in such identification. Nevertheless, we believe that the variability between groups and the lack of some relevant clinical information is probably minimalized, if not nullified, by the large sample size provided by the registry.

Furthermore, comorbidities were identified using ICD-10 codes and were treated as binary variables. Unfortunately, we were unable to assess the severity or control status (e.g., compensated vs. decompensated heart failure), which may influence outcomes but is not captured in the administrative data set.

Another limitation is the lack of standardization across centers. The NIS includes hospitals of varying sizes, geographies, and protocols, which introduces variability in clinical decision-making, timing of surgery, and documentation practices.

Despite these limitations, this study also has notable strengths. To the best of our knowledge, this is the largest analysis of ASBO to date, with about a million patients. NIS offers a comprehensive evaluation of both age and chronic health conditions as predictors of NOM failure across a wide and representative patient population. Importantly, our findings provide clinicians with practical, generalizable insights into the role of comorbidity burden in clinical decision-making for ASBO. Future studies should focus on prospective validation of predictive models incorporating age and comorbidity burden and the development of clinical decision-support tools to guide early intervention in patients at high risk of NOM failure.

## 6. Conclusions

Being aged over 65 years and the presence of chronic health conditions, excluding diabetes mellitus, are independent predictors of NOM failure in patients with ASBO. The presence of multiple comorbidities has an additive, cumulative effect on the risk of requiring surgery. These findings suggest that patient age and comorbidity profile should be incorporated into early risk stratification and management planning

## Figures and Tables

**Table 1:** Distribution of chronic health conditions by age group.

	Group A	Group B	*p* Value
DM	10,880 (1.13%)	8880 (1.35%)	<0.01
CHF	14,310 (1.49%)	30,620 (4.68%)	<0.01
CPD	54,500 (5.69%)	58,335 (8.91%)	<0.01
CKD	23,500 (2.45%)	38,925 (5.94%)	<0.01
PVD	14,600 (1.52%)	25,200 (3.85%)	<0.01

Numbers in the table represent isolated disease only. CHF—congestive heart failure; CKD—chronic kidney disease; CPD—chronic pulmonary disease; PVD—peripheral vascular disease; DM—diabetes mellitus.

**Table 2:** Failure of non-operative management (NOM) by comorbidity and age group.

		Group A	Group B	*p* Value
DM	Surgery	2375 (21.83%)	2050 (23.08%)	0.035
	No surgery	8505 (78.17%)	6830 (76.92%)	
CHF	Surgery	3360 (23.48%)	8405 (27.45%)	<0.01
	No surgery	10,950 (76.52%)	22,215 (72.55%)	
CPD	Surgery	13,040 (23.92%)	16,285 (27.91%)	<0.01
	No surgery	41,460 (76.08%)	42,050 (72.09%)	
CKD	Surgery	5545 (23.6%)	10,200 (26.2%)	<0.01
	No surgery	17,955 (76.4%)	28,725 (73.8%)	
PVD	Surgery	4555 (31.19%)	7835 (31.09%)	0.824
	No surgery	10,045 (68.81%)	17,365 (68.91%)	

CHF—congestive heart failure; CKD—chronic kidney disease; CPD—chronic pulmonary disease; PVD—peripheral vascular disease; DM—diabetes mellitus.

**Table 3:** Multivariable logistic regression model identifying independent predictors of operative intervention.

	OR	95% CI		*p* Value
		Lower	Upper	
Age > 65 year	1.387	1.377	1.398	<0.01
DM	1.014	0.981	1.049	0.405
CHF	1.158	1.113	1.183	<0.01
CPD	1.209	1.192	1.226	<0.01
CKD	1.121	1.101	1.142	<0.01
PVD	1.5	1.468	1.533	<0.01

CHF—congestive heart failure; CKD—chronic kidney disease; CPD—chronic pulmonary disease; PVD—peripheral vascular disease; DM—diabetes mellitus.

**Table 4:** Risk of NOM failure based on cumulative number of comorbidities, stratified by age group.

-	OR	95% CI		*p* Value
		Lower	Upper	
Group A				
1 comorbidity	1.382	1.362	1.402	<0.01
2 comorbidities	1.337	1.304	1.371	<0.01
3 comorbidities	1.464	1.399	1.533	<0.01
4+ comorbidities	1.562	1.418	1.721	<0.01
Group B				
1 comorbidity	1.142	1.127	1.157	<0.01
2 comorbidities	1.136	1.116	1.156	<0.01
3 comorbidities	1.213	1.181	1.245	<0.01
4+ comorbidities	1.144	1.088	1.203	<0.01

## Data Availability

All data was taken from the National (Nationwide) Inpatient Sample (NIS). This database is part of a family of databases and software tools developed for the Healthcare Cost and Utilization Project (HCUP). The NIS is the largest publicly available all-payer inpatient healthcare database designed to produce U.S. regional and national estimates of inpatient utilization, access, cost, quality, and outcomes. Unweighted, it contains data from around 7 million hospital stays each year. Weighted, it estimates around 35 million hospitalizations nationally. Developed through a Federal-State-Industry partnership sponsored by the Agency for Healthcare Research and Quality (AHRQ), HCUP data inform decision-making at the national, state, and community levels.

## Abbreviations

ASBO	adhesive small bowel obstruction
NOM	non-operative management
DM	diabetes mellitus
CHF	congestive heart failure
CKD	chronic kidney disease
CPD	chronic pulmonary disease
